# How do GPs Want Large Language Models to be Applied in Primary Care, and What Are Their Concerns? A Cross‐Sectional Survey

**DOI:** 10.1111/jep.70129

**Published:** 2025-05-14

**Authors:** Richard C. Armitage

**Affiliations:** ^1^ Academic Unit of Population and Lifespan Sciences, School of Medicine University of Nottingham Nottingham UK

## Abstract

**Introduction:**

Although the potential utility of large language models (LLMs) in medicine and healthcare is substantial, no assessment has been made to date of how GPs want LLMs to be applied in primary care, or of which issues GPs are most concerned about regarding the implementation of LLMs into their clinical practice. This study's objective was to generate preliminary evidence that answers these questions, which are relevant because GPs themselves will ultimately harness the power of LLMs in primary care.

**Methods:**

Non‐probability sampling was utilised: GPs practicing in the UK and who were members of one of two Facebook groups (one containing a community of UK primary care staff, the other containing a community of GMC‐registered doctors in the UK) were invited to complete an online survey, which ran from 06 to 13 November 2024.

**Results:**

The survey received 113 responses, 107 of which were from GPs practicing in the UK. When LLM accuracy and safety were assumed to be guaranteed, broad enthusiasm for LLMs carrying out various nonclinical and clinical tasks in primary care was reported. The single nonclinical task and clinical task that respondents were most supportive of were the LLM listening to the consultation and writing notes in real‐time for the GP to review, edit, and save (44.0%), and the LLM identifying outstanding clinical tasks and actioning them (51.0%), respectively. Respondents were concerned with a range of issues regarding LLMs being embedded into clinical systems, with patient safety being the most commonly reported single issue of concern (36.2%).

**Discussion:**

This study has generated preliminary evidence that is of potential utility to those developing LLMs for use in primary care. Further research is required to expand this evidence base to further inform the development of these technologies, and to ensure they are acceptable to the GPs who will use them.

## 1 Introduction

Large language models (LLMs) are a type of generative artificial intelligence (AI) that are trained on large amounts of textual data. By identifying structures and patterns in human language, LLMs can generate novel human‐like text in response to textual prompts. LLMs that are currently available for public use include GPT (OpenAI), Gemini (Google), Llama (Meta), Claude (Anthropic), Phi‐3 (Microsoft), and Grok (xAI).

The potential applications of LLMs in medicine and healthcare are being increasingly explored. GPT‐4 has been shown to perform strongly when answering postgraduate medical examination questions across various specialities including general practice [1], paediatrics [2], surgery [3], and women's health [4]. With this foundational ‘knowledge,’ there is great potential for LLMs to assist clinicians in their clinical practice [5, 6], including in specific clinical specialities such as primary care [7], cardiology [8], and ophthalmology [9]. More broadly, the utility of LLMs has been recognised in medical education [10], generating scholarly content [11], contributing to peer review [12], parsing unstructured clinical notes [13], proving antimicrobial prescribing advice [14], and writing discharge summaries [15], clinic letters [16], and simplified radiology reports [17].

The ethical, legal, and regulatory issues associated with the use of LLMs in clinical practice have been well described [18, 19, 20, 21, 22]. To date, no LLM has been granted authorisation for use in clinical practice, including in primary care. Despite this, 20% of GPs in the UK who responded to a recent survey reported using generative AI tools in their practice [23]. It is clear, therefore, that many GPs recognise the potential for LLMs to assist them in their clinical practice. However, to the author's knowledge, no assessment has been made to date of specifically how GPs want LLMs to be applied in primary care, or of which issues GPs are most concerned about regarding the implementation of LLMs into their practice. These are highly relevant questions because it is GPs themselves who will ultimately harness the power of LLMs in clinical practice, meaning the preferences and professional, practical, and tacit knowledge of practicing GPs are vital to inform the development and deployment of LLMs that are maximally useful to GPs, acceptable to them, and harmonised with their clinical workflows. The objective of this study is to begin to answer these questions, specifically amongst currently practicing GPs.

## 2 Methods

An online survey was produced using the Google Forms platform and was pre‐tested and piloted with three UK GPs. Non‐probability sampling was utilised: GPs who are currently practicing in the UK were invited to complete the survey via one post on two Facebook groups: one containing a community of primary care staff in the UK (containing approximately 24,500 members during the survey period), and another containing a community of General Medical Council registered doctors in the UK (containing approximately 16,300 members during the survey period). No financial incentives were offered. All participants gave informed consent before taking part. The first question, which was mandatory to complete, asked respondents whether they are a practising GP in the UK (see Supporting Information 1)? The survey was open from 06 November 2024 to 13 November 2024.

## 3 Results

There were 113 responses to the survey, 107 of which were from currently practicing GPs in the UK. Only responses from currently practicing GPs in the UK were analysed. 90.7% (97) were aware of LLMs before completing the survey, and 68.2% (73) had personally used a LLM (note the question did not refer to using a LLM in clinical practice).

Participants were asked to imagine that an LLM is embedded into their clinical system, and to assume that the LLM's accuracy and safety are guaranteed. When asked which nonclinical tasks would be useful for the LLM to perform (104 responses received), 86.5% (90) reported the LLM listening to the consultation and interpreting aloud in real‐time (if the patient did not speak English), 81.7% (85) reported the LLM reviewing the patient's clinical record and presenting a summary for the GP to read before seeing the patient, 79.8% (83) reported the LLM listening to the consultation and writing notes in real‐time for the GP to review, edit, and save, and 6.7% (7) reported a different application. When asked to select which of these nonclinical applications would be the most useful (100 responses received), the most common response (44.0%) was the LLM listening to the consultation and writing notes in real‐time for the GP to review, edit, and save. When asked which clinical tasks would be useful for the LLM to perform (109 responses received), 89.9% (98) reported the LLM identifying outstanding clinical tasks (e.g. annual U&E) and actioning them (e.g. inviting the patient for a blood test), 56.9% (62) reported the LLM actioning clinical administrative tasks (e.g. letters and blood results), 53.2% (58) reported the LLM conducting routine medication reviews, 43.1% (47) reported the LLM listening to the consultation and suggesting differential diagnoses for the GP to consider, 33.9% (37) reported the LLM listening to the consultation and suggesting management plans for the GP to consider, 27.5% (30) reported the LLM triaging patients, and 3.7% (4) reported a different application. When asked to select which of these clinical applications would be the most useful (101 responses received), the most common response (51.0%) was the LLM identifying outstanding clinical tasks (e.g. annual U&E) and actioning them (e.g. inviting the patient for a blood test).

Participants were asked to imagine that an LLM is embedded into their clinical system. When asked which issues they would be concerned about with regard to the LLM (107 responses received), 88.8% (95) reported clinical liability, 86.9% (93) reported accuracy, 79.4% (85) reported patient safety, 54.2% (58) reported harm to the doctor‐patient relationship, 40.2% (43) reported integration with clinical workflows, and 7.5% (8) reported a different issue. When asked to select which of these issues would be the most concerning (105 responses received), the most common response (36.2%) was patient safety (see Supporting Information 2).

## 4 Discussion

To the author's knowledge, this is the first attempt to formally assess how GPs want LLMs to be applied in primary care, and to assess which issues GP are most concerned about regarding the implementation of LLMs into their practice. When LLM accuracy and safety are assumed to be guaranteed, the results demonstrate broad enthusiasm amongst GPs for LLMs to carry out various nonclinical and clinical tasks in primary care. The single nonclinical task and clinical task that respondents were most supportive of was the LLM listening to the consultation and writing notes in real‐time for the GP to review, edit, and save, and the LLM identifying outstanding clinical tasks (e.g. annual U&E) and actioning them (e.g. inviting the patient for a blood test), respectively. However, GPs are concerned with a range of issues regarding LLMs being embedded into their clinical systems, with patient safety being the most commonly reported single issue of concern.

This study has limitations. First, the number of respondents was small relative to the 38,420 full‐time equivalent GPs currently practicing in England alone [24]. Second, due to the convenience‐based sampling strategy, a non‐probability sample of GPs who were active members of the two Facebook groups in which the invitation was posted was generated. Accordingly, the survey may not be representative of GPs practicing in the UK, and the results cannot be generalised to this population. Third, demographic data, such as sex, age, geographical location within the UK, and length of clinical experience, were not collected, thereby compounding the limitation that the survey may not be representative of GPs practicing in the UK. Fourth, the decision to participate in the survey or not might have introduced selection biases, such as if those with a favourable disposition towards generative AI in primary care were more likely to participate than those with an oppositional stance.

Considering these limitations, this study has generated preliminary evidence that is of potential utility to those aiming to develop LLMs for deployment and widespread adoption by GPs in primary care. Further research is urgently required to expand this evidence base – including larger quantitative studies (from a wider range of sources, and that includes participants' demographic characteristics) and qualitative research (in the form of focus groups) regarding GP opinions on this matter – to inform the development of LLMs intended for deployment in primary care, and to ensure that these technologies will be acceptable to the GPs who will eventually use them.

## Conflicts of Interest

The author declares no conflict of interest.

## Supporting information

Supplemental Material 1.

Supplemental Material 2.

## Data Availability

The data that supports the findings of this study are available in the Supporting Material of this article.

## References

[1] R. Armitage , “Performance of Generative Pre‐Trained Transformer‐4 (GPT‐4) in Membership of the Royal College of General Practitioners (MRCGP)‐Style Examination Questions,” Postgraduate Medical Journal 100, no. 1182 (April 2024): 274–275, 10.1093/postmj/qgad128. 38142282

[2] R. Armitage , “Performance of GPT‐4 in Membership of the Royal College of Paediatrics and Child Health‐Style Examination Questions,” BMJ Paediatrics Open 8, no. 1 (2024): e002575, 10.1136/bmjpo-2024-002575. 38508662 PMC10961496

[3] J. Chan , T. Dong , and G. Angelini , “The Performance of Large Language Models in Intercollegiate Membership of the Royal College of Surgeons Examination,” Annals of The Royal College of Surgeons of England 106, no. 8 (November 2024): 700–704, 10.1308/rcsann.2024.0023. 38445611 PMC11528401

[4] R. Armitage , “Performance of Generative Pre‐Trained Transformer‐4 (GPT‐4) in RCOG Diploma (DRCOG)‐Style Questions,” Postgraduate Medical Journal 100, no. 1187 (September 2024): 695–696, 10.1093/postmj/qgae038. 38497288

[5] J. Clusmann , F. R. Kolbinger , H. S. Muti , et al., “The Future Landscape of Large Language Models in Medicine,” Communications Medicine 3 (October 2023): 141, 10.1038/s43856-023-00370-1. 37816837 PMC10564921

[6] A. J. Thirunavukarasu , D. S. J. Ting , K. Elangovan , L. Gutierrez , T. F. Tan , and D. Ting , “Large Language Models in Medicine,” Nature Medicine 29 (2023): 1930–1940, 10.1038/s41591-023-02448-8. 37460753

[7] A. Andrew , “Potential Applications and Implications of Large Language Models in Primary Care,” Family Medicine and Community Health 12 (January 2024): e002602, 10.1136/fmch-2023-002602. 38290759 PMC10828839

[8] G. Quer and E. J. Topol , “The Potential for Large Language Models to Transform Cardiovascular Medicine,” Lancet Digital Health 6, no. 10 (October 2024): e767–e771, 10.1016/S2589-7500(24)00151-1. 39214760

[9] B. K. Betzler , H. Chen , C. Y. Cheng , et al., “Large Language Models and Their Impact in Ophthalmology,” Lancet Digital Health 5, no. 12 (December 2023): e917–e924, 10.1016/S2589-7500(23)00201-7. 38000875 PMC11003328

[10] R. C. Armitage , “Chatgpt: The Threats to Medical Education,” Postgraduate Medical Journal 99, no. 1176 (September 2023): 1130–1131, 10.1093/postmj/qgad046. 37410672

[11] M. Liebrenz , R. Schleifer , A. Buadze , D. Bhugra , and A. Smith , “Generating Scholarly Content With ChatGPT: Ethical Challenges for Medical Publishing,” Lancet Digital Health 5, no. 3 (March 2023): e105–e106, 10.1016/S2589-7500(23)00019-5. 36754725

[12] T. Donker , “The Dangers of Using Large Language Models for Peer Review,” Lancet Infectious Diseases 23, no. 7 (July 2023): 781, 10.1016/S1473-3099(23)00290-6. 37178707

[13] A. Vaid , I. Landi , G. Nadkarni , and I. Nabeel , “Using Fine‐Tuned Large Language Models to Parse Clinical Notes in Musculoskeletal Pain Disorders,” Lancet Digital Health 5, no. 12 (December 2023): e855–e858, 10.1016/S2589-7500(23)00202-9. 39492289

[14] A. Howard , W. Hope , and A. Gerada , “ChatGPT and Antimicrobial Advice: The End of the Consulting Infection Doctor?,” Lancet Infectious Diseases 23, no. 4 (April 2023): 405–406, 10.1016/S1473-3099(23)00113-5. 36822213

[15] S. B. Patel and K. Lam , “ChatGPT: The Future of Discharge Summaries?,” Lancet Digital Health 5, no. 3 (March 2023): e107–e108, 10.1016/S2589-7500(23)00021-3. 36754724

[16] S. R. Ali , T. D. Dobbs , H. A. Hutchings , and I. S. Whitaker , “Using ChatGPT to Write Patient Clinic Letters,” Lancet Digital Health 5, no. 4 (April 2023): e179–e181, 10.1016/S2589-7500(23)00048-1. 36894409

[17] K. Jeblick , B. Schachtner , J. Dexl , et al, “ChatGPT Makes Medicine Easy to Swallow: An Exploratory Case Study on Simplified Radiology Reports,” European Radiology 34 (October 2023): 2817–2825, 10.1007/s00330-023-10213-1. 37794249 PMC11126432

[18] J. C. L. Ong , S. Y. H. Chang , W. William , et al., “Ethical and Regulatory Challenges of Large Language Models in Medicine,” Lancet Digital Health 6, no. 6 (June 2024): e428–e432, 10.1016/S2589-7500(24)00061-X. 38658283

[19] H. Li , J. T. Moon , S. Purkayastha , L. A. Celi , H. Trivedi , and J. W. Gichoya , “Ethics of Large Language Models in Medicine and Medical Research,” Lancet Digital Health 5, no. 6 (June 2023): e333–e335, 10.1016/S2589-7500(23)00083-3. 37120418

[20] S. Harrer , “Attention Is Not All You Need: The Complicated Case of Ethically Using Large Language Models in Healthcare and Medicine,” EBioMedicine 90 (April 2023): 104512, 10.1016/j.ebiom.2023.104512. 36924620 PMC10025985

[21] R. Armitage , “Implications of Large Language Models for Clinical Practice: Ethical Analysis Through the Principlism Framework,” Journal of Evaluation in Clinical Practice 90: 104512, 10.1111/jep.14250. PMC11609490 39618089

[22] B. Meskó and E. J. Topol , “The Imperative for Regulatory Oversight of Large Language Models (Or Generative AI) in Healthcare,” npj Digital Medicine 6 (2023): 120, 10.1038/s41746-023-00873-0. 37414860 PMC10326069

[23] C. R. Blease , C. Locher , J. Gaab , M. Hägglund , and K. D. Mandl , “Generative Artificial Intelligence in Primary Care: An Online Survey of UK General Practitioners,” BMJ Health & Care Informatics 31 (2024): e101102, 10.1136/bmjhci-2024-101102. PMC11429366 39288998

[24] NHS England . General Practice Workforce, 30 September 2024, accessed November 13, 2024, https://digital.nhs.uk/data-and-information/publications/statistical/general-and-personal-medical-services/30-september-2024.

