# Supplementary material for: How do GPs Want Large Language Models to be Applied in Primary Care, and What Are Their Concerns? A Cross‐Sectional Survey
Source: J Eval Clin Pract. 2025 May 14;31(4):e70129. doi: 10.1111/jep.70129 (PMC12079004; doi:10.1111/jep.70129)
Supplement: Supplementary file 1 — Supplemental Material 1. [file JEP-31-0-s002.docx]

**Survey questions**

----------------------------------------------------------------------------------------------------------------

Screen 1

Title: The potential roles of large language models in primary care: GP opinions

You have been invited to complete this survey as you are a practicing GP in the UK.

The survey aims to collect your opinions on the potential roles of large language models in primary care.

It should take less than 5 minutes to complete.

- Are you a practicing GP in the UK?*
  - *Yes*
  - *No*

* Answer is required

----------------------------------------------------------------------------------------------------------------

Screen 2

A large language model (LLM) is a type of artificial intelligence (AI) that can analyse and generate human language.

LLMs are trained on vast amounts of data, such as books and articles, to learn the statistical relationships between words and phrases.  This allows them to perform tasks such as translation, question answering, and text completion.

Examples of LLMs are ChatGPT (from OpenAI), Gemini (from Google), Claude (from Anthropic), and Llama (from Meta).

- Were you aware of large language models prior to completing this survey?
- Have you personally used a large language model such as ChatGPT, Gemini, Claude, or Llama?

----------------------------------------------------------------------------------------------------------------

Screen 3

- Imagine a large language model is embedded into your clinical system (e.g. EMIS Web or SystmOne). If accuracy and safety are guaranteed, which potential roles of the large language model would you find useful? Tick all that apply.

*Non-clinical tasks:*

- - *The LLM reviews the patient’s clinical record and presents a summary for you to read before you see the patient.*
  - *The LLM listens to the consultation and writes notes in real-time for you to review, edit, and save.*
  - *If the patient does not speak English, the LLM listens to the consultation and interprets aloud in real-time (replacing the need for a human interpreter).*
  - *Other*

*Clinical tasks:*

- - *The LLM triages patients.*
  - *The LLM listens to the consultation and suggests differential diagnoses for you to consider.*
  - *The LLM listens to the consultation and suggests management plans for you to consider.*
  - *The LLM identifies outstanding clinical tasks (e.g. annual U&E) and actions them (e.g. invites the patient for a blood test).*
  - *The LLM conducts routine medication reviews.*
  - *The LLM actions clinical administrative tasks (e.g. letters and blood results).*
  - *Other*

----------------------------------------------------------------------------------------------------------------

Screen 4

- Imagine a large language model is embedded into your clinical system (e.g. EMIS Web or SystmOne). If accuracy and safety are guaranteed, which potential role of the large language model would you find most useful? Select one.

*Non-clinical tasks:*

- - *The LLM reviews the patient’s clinical record and presents a summary for you to read before you see the patient.*
  - *The LLM listens to the consultation and writes notes in real-time for you to review, edit, and save.*
  - *If the patient does not speak English, the LLM listens to the consultation and interprets aloud in real-time (replacing the need for a human interpreter).*
  - *Other.*

*Clinical tasks:*

- - *The LLM triages patients.*
  - *The LLM listens to the consultation and suggests differential diagnoses for you to consider.*
  - *The LLM listens to the consultation and suggests management plans for you to consider.*
  - *The LLM identifies outstanding clinical tasks (e.g. annual U&E) and actions them (e.g. invites the patient for a blood test).*
  - *The LLM conducts routine medication reviews.*
  - *The LLM actions clinical administrative tasks (e.g. letters and blood results).*
  - *Other.*

----------------------------------------------------------------------------------------------------------------

Screen 5

- If a large language model is embedded into your clinical system (e.g. EMIS Web or SystmOne), which of the following would you be concerned about with regard to the large language model? Tick all that apply.
- *Accuracy*
- *Patient safety*
- *Clinical liability*
- *Integration with clinical workflows*
- *Harm to the doctor-patient relationship*
- *Other*

----------------------------------------------------------------------------------------------------------------

Screen 6

- If a large language model is embedded into your clinical system (e.g. EMIS Web or SystmOne), which of the following would you be most concerned about with regard to the large language model? Select one.
- *Accuracy*
- *Patient safety*
- *Clinical liability*
- *Integration with clinical workflows*
- *Harm to the doctor-patient relationship*
- *Other*

----------------------------------------------------------------------------------------------------------------
